# Previously unknown evolutionary groups dominate the ssDNA gokushoviruses in oxic and anoxic waters of a coastal marine environment

**DOI:** 10.3389/fmicb.2015.00315

**Published:** 2015-04-22

**Authors:** Jessica M. Labonté, Steven J. Hallam, Curtis A. Suttle

**Affiliations:** ^1^Department of Microbiology and Immunology, University of British Columbia, Vancouver, BCCanada; ^2^Canadian Institute for Advanced Research, Toronto, ONCanada; ^3^Graduate Program in Bioinformatics, University of British Columbia, Vancouver, BCCanada; ^4^Department of Earth, Ocean and Atmospheric Sciences, University of British Columbia, Vancouver, BCCanada; ^5^Department of Botany, University of British Columbia, Vancouver, BCCanada

**Keywords:** Gokushovirinae, host range, viral diversity, SUP05, oxygen minimum zones, PCR sequencing

## Abstract

Metagenomic studies have revealed that ssDNA phages from the family *Microviridae* subfamily *Gokushovirinae* are widespread in aquatic ecosystems. It is hypothesized that gokushoviruses occupy specialized niches, resulting in differences among genotypes traversing water column gradients. Here, we use degenerate primers that amplify a fragment of the gene encoding the major capsid protein to examine the diversity of gokushoviruses in Saanich Inlet (SI), a seasonally anoxic fjord on the coast of Vancouver Island, BC, Canada. Amplicon sequencing of samples from the mixed oxic surface (10 m) and deeper anoxic (200 m) layers indicated a diverse assemblage of gokushoviruses, with greater richness at 10 m than 200 m. A comparison of amplicon sequences with sequences selected on the basis of RFLP patterns from eight surface samples collected over a 1-year period revealed that gokushovirus diversity was higher in spring and summer during stratification and lower in fall and winter after deep-water renewal, consistent with seasonal variability within gokushovirus populations. Our results provide persuasive evidence that, while specific gokushovirus genotypes may have a narrow host range, hosts for gokushoviruses in SI consist of a wide range of bacterial taxa. Indeed, phylogenetic analysis of clustered amplicons revealed at least five new phylogenetic groups of previously unknown sequences, with the most abundant group associated with viruses infecting SUP05, a ubiquitous and abundant member of marine oxygen minimum zones. Relatives of SUP05 dominate the anoxic SI waters where they drive coupled carbon, nitrogen, and sulfur transformations along the redoxline; thus, gokushoviruses are likely important mortality agents of these bacteria with concomittant influences on biogeochemical cycling in marine oxygen minimum zones.

## Introduction

Bacteriophages belonging to the *Microviridae* family consist of a ∼30 nm icosahedral capsid containing a positive-sense ssDNA molecule of 4.5–6.1 kb (King et al., 2012). Replication requires a minimum of two coat proteins (VP1 and VP2), a scaffolding protein (VP3), a replication protein (VP4), and a DNA packaging protein (VP5). Based on the phylogeny of the major capsid protein (MCP or VP1) from isolates, the *Microviridae* family is divided into two groups (Brentlinger et al., 2002); the *Microvirus* genus contains phages like phiX174 and G4 that infect *Escherichia coli* (Godson et al., 1978), while the *Gokushovirinae* subfamily includes those infecting parasitic bacteria such as *Chlamydia* [Chp1 (Storey et al., 1989), Chp2 (Liu et al., 2000; Everson et al., 2002), Chp3 (Garner et al., 2004)], *Bdellovibrio* [(phiMH2K; Brentlinger et al., 2002)], and *Spiroplasma* [(SpV4; Chipman et al., 1998)]. While it is commonly thought that *Microviridae* phages are strictly lytic (Liu et al., 2000; Garner et al., 2004; Salim et al., 2008), an *in silico* study found sequences with similar genome organization to gokushoviruses associated with *Bacteroidetes* from the human gut and mouth, which suggests that these phages can be temperate (Krupovic and Forterre, 2011). The temperate *Microviridae* phages are phylogenetically distinct from gokushoviruses, and have been assigned to the *Alpavirinae*, a proposed new sub-family within the *Microviridae* (Krupovic and Forterre, 2011). Recently, 81 microvirus genomes (including 42 gokushoviruses) were assembled from various environmental metegenomic data, identifying a new group, the *Pichovirinae*, which harbored a different genome organization of the conserved genes, indicating that microviruses display great diversity and may play an important role in many ecosystems (Roux et al., 2012b).

Marine gokushoviruses were first revealed in viral metagenomic data from the Strait of Georgia (SOG), Gulf of Mexico (GOM), and Sargasso Sea (SAR; Angly et al., 2006), and are among the most commonly recovered sequences from ssDNA phages in marine metagenomic data (Rosario and Breitbart, 2011). They were particularly abundant in the SAR, where 6% of the sequences were similar to the phage Chp1 that infects *Chlamydia psittaci* (Angly et al., 2006). This abundance of ssDNA sequences allowed for the assembly of two environmental *Gokushovirinae* genomes, with the help of PCR amplification (Tucker et al., 2011). A survey in the Atlantic Ocean showed a different depth distribution of these two genomes, consistent with alternative host-infection patterns. Sequences belonging to gokushoviruses have also been found in marine (Labonté and Suttle, 2013b; McDaniel et al., 2014) and fresh waters (López-Bueno et al., 2009; Roux et al., 2012a), stromatolites (Desnues et al., 2008), confined aquifers (Smith et al., 2013), and pelagic sediments (Yoshida et al., 2013). Based on these observations, degenerate primers designed to amplify fragments of the genes encoding the replication initiator (ORF4 or Rep; Tucker et al., 2011) and MCP (Labonté and Suttle, 2013a; Hopkins et al., 2014) were used to examine the distribution and diversity of gokushoviruses in marine ecosystems. Phylogenetic analyses of the amplified fragments revealed that ssDNA phages have different geographic distributions (Labonté and Suttle, 2013a), and that the genetic distance of gokushovirus sequences increased with geographic distance (Tucker et al., 2011). Most of the hosts of environmental gokushoviruses are unknown, but it is hypothesized that they occupy specialized niches, and that specific gokushovirus genotypes have limited geographic range (Angly et al., 2006; Tucker et al., 2011; Labonté and Suttle, 2013a).

Saanich Inlet (SI) is a steep-sided fjord with restricted circulation due to a shallow glacial sill located at the entrance. During spring and summer, high primary productivity in surface waters combined with limited basin circulation contribute to the formation of deep-water anoxia (Anderson, 1973). The anoxic zone is characterized by accumulation of CH_4_, NH_3_, and H_2_S (Anderson, 1973; Lilley et al., 1982; Ward et al., 1989). Typically, in late summer oxygenated nutrient-rich water from Haro Strait (connecting SI to the SOG) cascades over the sill, mixing the oxic, and anoxic waters from top to bottom (Anderson, 1973). Recently, single-cell amplified genomic data (SAGs) from uncultured SUP05 bacteria from marine oxygen minimum zones revealed identical *Microviridae* sequences in 8 of 127 SAGs, suggesting a recent infection event (Roux et al., 2014).

Here, rather than looking at seasonal changes (Labonté and Suttle, 2013a), the diversity of gokushoviruses was examined in the mixed oxic surface (10 m) and deeper anoxic (200 m) layers of SI to better understanding their dynamics and roles in environments with contrasting levels of oxygen. We used degenerate primers for the MCP in combination with amplicon sequencing using 454 technology to reveal a diverse assemblage of gokushoviruses with greater richness at 10 m than 200 m. The results provide persuasive evidence that gokushoviruses likely infect a wide range of hosts, and may be important mortality agents of the SUP05 clade of gamma proteobacteria, an important taxonomic group involved in carbon, nitrogen, and sulfur cycling in marine oxygen minimum zones.

## Materials and Methods

### Preparation of Marine Samples

**FIGURE 1:**
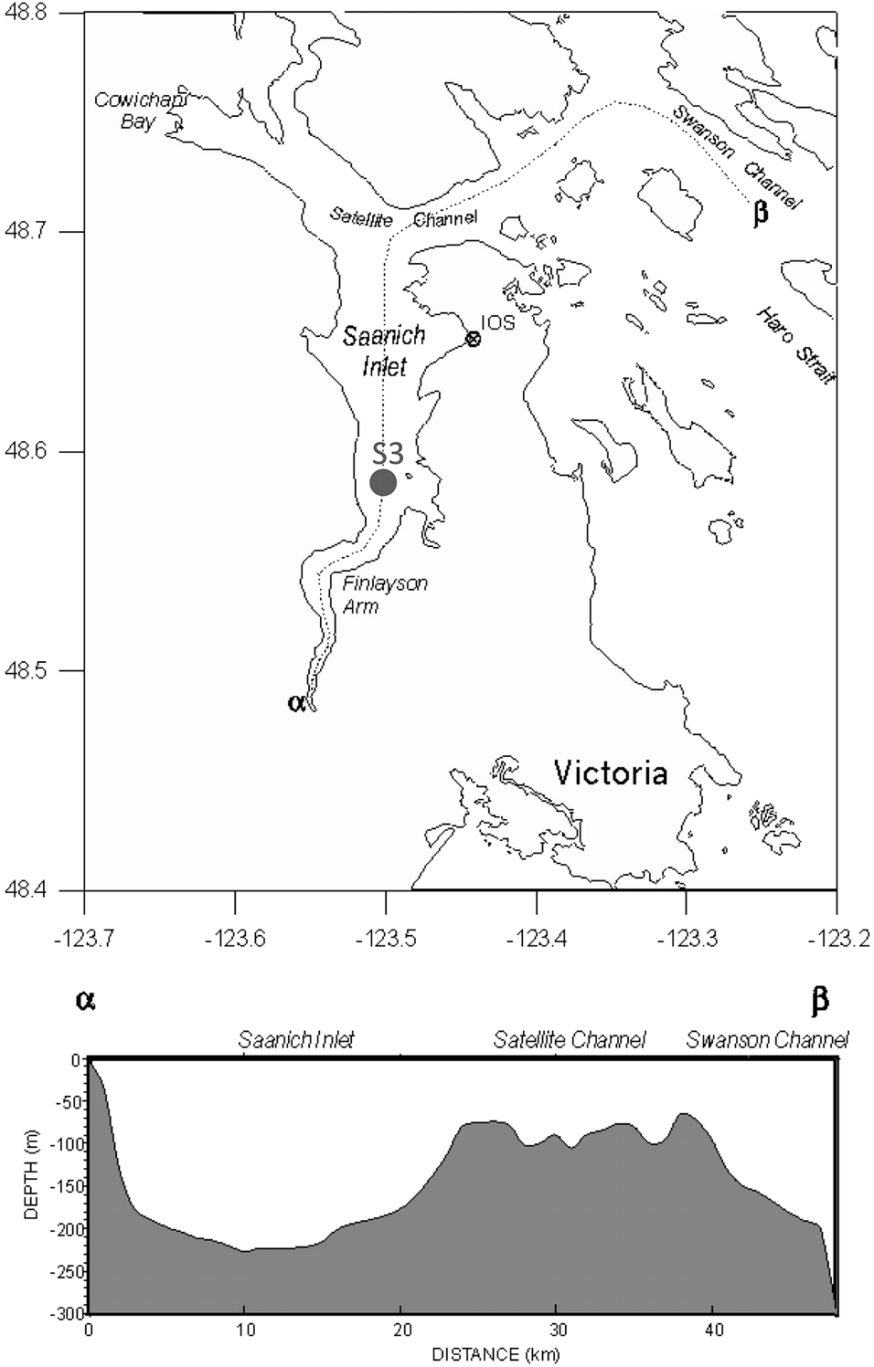
**Map of Saanich Inlet (SI; **top)** and cross-sectional depth profile (bottom)**. Station S3 (48°35′N, 123°30′W) was sampled in the current study. Map adapted from Fisheries and Oceans Canada (http://www.pac.dfo-mpo.gc.ca/science/oceans/BCinlets/saanich-eng.htm).

On a monthly basis, ∼20 L of water from Station S3 in SI (**Figure 1**) were filtered to remove cells using 0.22-μm pore-size Sterivex^TM^ filter units (Millipore). The viruses were concentrated from the filtrate by tangential flow filtration using a TFF 30-kDa cartridge (Millipore) to a final volume of ∼250 mL, and stored at 4°C until used following the procedure outlined in (Suttle et al., 1991). Amplicons were sequenced from two composite samples of virus concentrates comprised of samples collected from 10 m and 200 m, respectively, during April 2007, and February, March, April, June, August, and November (200 m), or December (10 m) 2008.

### ssDNA Purification, PCR Amplification, and Sequencing

For each virus concentrate, viral ssDNA was extracted using a QIAprep Spin M13 kit (Qiagen), according to the manufacturer’s protocol. To create double-stranded DNA, 5 μL of the ssDNA preparation was subjected to multiple displacement amplification (MDA; Repli-g Mini kit), and purified using a QIAamp DNA Mini kit. Denaturation of dsDNA was limited during MDA by adding the stop solution N1 immediately after the denaturation solution D1. The purified MDA DNA was resuspended in 100 μL of TE, and 10 μL was used as template in each PCR reaction mixture consisting of Taq DNA polymerase assay buffer [20 mM Tris⋅HCl (pH 8.4), 50 mM KCl], 1.5 mM MgCl2, 125 μM of each deoxyribonucleoside triphosphate, 1 μM of each primer amplifying a fragment of the MCP gene [MicroVP1–F1 (CGN GCN TAY AAY TTR ATH), VP1–F2 (AGN GCN TAY AAY TTR CTN), MicroVP1–R1 (NCG YTC YTG RTA NCC RAA), and Micro-VP1–R2 (NCT YTC YTG RTA NCC RAA)] and 2.5 U of Platinum^®^ Taq DNA polymerase (Invitrogen). Forward and reverse primer pairs were used to reduce the degeneracy within a single primer. Negative controls contained all reagents except DNA template. The samples were denatured at 94°C for 3 min, followed by 35 cycles of denaturation at 94°C for 30 s, annealing at 50°C for 30 s, and elongation at 72°C for 50 s, with a final elongation step of 72°C for 5 min. The expected PCR product was ∼800 bp in length. PCR amplicons were purified with a MinElute PCR purification kit (Qiagen), pooled into mixes from the 10 or 200 m depth and concentrated using a Millipore YM-30 Microcon centrifugal filter to a final volume of ∼50 μL; a total of 500 ng of DNA amplicons from each pooled 10 and 200 m sample were sent for pyrosequencing using Roche 454 FLX instrumentation with Titanium chemistry at the Broad Institute at the Massachusetts Institute of Technology.

### Sequence Binning and Clustering

The sequences were screened for quality and length. Reads were removed if they contained one or more ambiguous bases (Ns), were shorter than 200 nucleotides, or did not match the priming site at the proximal end. Reads were binned based on the primer sequence, which was subsequently trimmed. Sequences arising from both forward primers (F) and both reverse primers (R) were combined resulting in F and R bins. Sequence errors can occur throughout the workflow, including an error rate of 1 per 10^6^–10^7^ bp (Dean et al., 2001) for WGA, and homopolymers, insertions and deletions of about 0.1% per base for pyrosequencing (Margulies et al., 2005; Huse et al., 2010; Quince et al., 2011); however, the impact of these errors were minimized by clustering the reads into operational taxonomic units (OTUs) at 95% identity using CD-hit (Li and Godzik, 2006). Clustering at 95% also recruited most singletons into an OTU and allowed the data to be compared with the OTUs from a previous seasonal study of gokushoviruses in SI (Labonté and Suttle, 2013a).

Operational taxonomic units were queried in a BLAST search analysis (NCBI BLAST 2.2.2) using an *e*-value cut-off of 10^-5^ against a manually curated database derived from environmental sequences and sequences from gokushovirus isolates composed of all the *Microviridae* genomes available in GenBank (as of November 23, 2014), assembled genomes from Roux et al. (2012b), environmental MCP amplicon sequences from Labonté and Suttle (2013a) and Hopkins et al. (2014). OTUs that did not have a significant hit to sequenced gokushoviruses were removed from the phylogenetic analysis.

### Diversity and Species Richness Calculations

For each primer bin, a rank-abundance distribution of phylotypes was generated and subsequently fitted to a power-law function using non-linear regression. For each primer bin, rarefaction species richness curves, and diversity indices were calculated using the Vegan Ecological Diversity package in R (R Development Core Team, 2011). Total estimated richness (*S_p_*) was calculated following Chao’s equation (Chao, 1987). The Shannon–Weaver (*H*′) diversity index was calculated as in Hill (1973) on a subsample of 700 reads.

### Phylogenetic Analyses

Nucleotide OTUs were aligned with other environmental sequences (Roux et al., 2012b; Yoshida et al., 2013; Hopkins et al., 2014) using MAFFT (Katoh et al., 2002) with the E-INS-I parameters. We worked with the nucleotide sequences because of problems such as homopolymers associated with the 454 platform made it difficult to accurately infer the correct amino-acid sequences. The alignment of the end product (R primers) was trimmed to get the conserved regions only and phylogenetic analysis was performed with phyML (Guindon et al., 2010) under the HKY85 substitution model with an invgamma distribution with approximate likelihood ratio test (aLRT). Trees were viewed in FigTree^1^.

### Nucleotide Sequence Accession

Raw sequences, OTU sequences, alignments, and trees are publicly available on Dryad^2^.

## Results and Discussion

The results from our analyses showed that the oxic and anoxic waters of SI are home to diverse gokushoviruses that comprise at least five previously unknown phylogenetic groups, composed of many numerically dominant OTUs. The most abundant group is associated with viruses that infect SUP05, a group of sulfur-oxidizing bacteria that are ubiquitous and abundant players in marine oxygen minimum zones. These results and their interpretations are detailed below.

### Amplicon Sequencing of the MCP From Gokushoviruses

Amplicon sequences of the MCP from two pooled mixes (10 and 200 m) generated 7195 (F) and 2135 (R) good reads (no N, exact primer match, no chimeras) for the 10-m bin and 2687 (F) and 710 (R) good reads for the 200-m bin (**Table 1**). Quality controls using gel electrophoresis and DNA quantification, indicated that the yields from PCR amplification of the 200-m samples were consistently lower than for the 10-m samples (data not shown), which can explain the lower number of MCP amplicons obtained for the 200-m samples.

**Table 1:** Number of reads and OTUs after clustering at 95% similarity and OTUs sharing similarity with known *Microviridae* phages.

	Primer	Number of good reads	Number of OTUs	Microviridae OTUs	Most abundant % reads	Singletons % reads	Doublets % reads
10 m	F	7195	504	446	17.9	2.9	2.1
	R	2135	228	213	16.2	4.3	3
200 m	F	2687	117	80	14.8	1.6	0.8
	R	710	36	25	21.5	1.7	1.1

*Clustering was performed on sequences that were >200 bp. The number of reads represents the number of reads without ambiguous base.*

**FIGURE 2:**
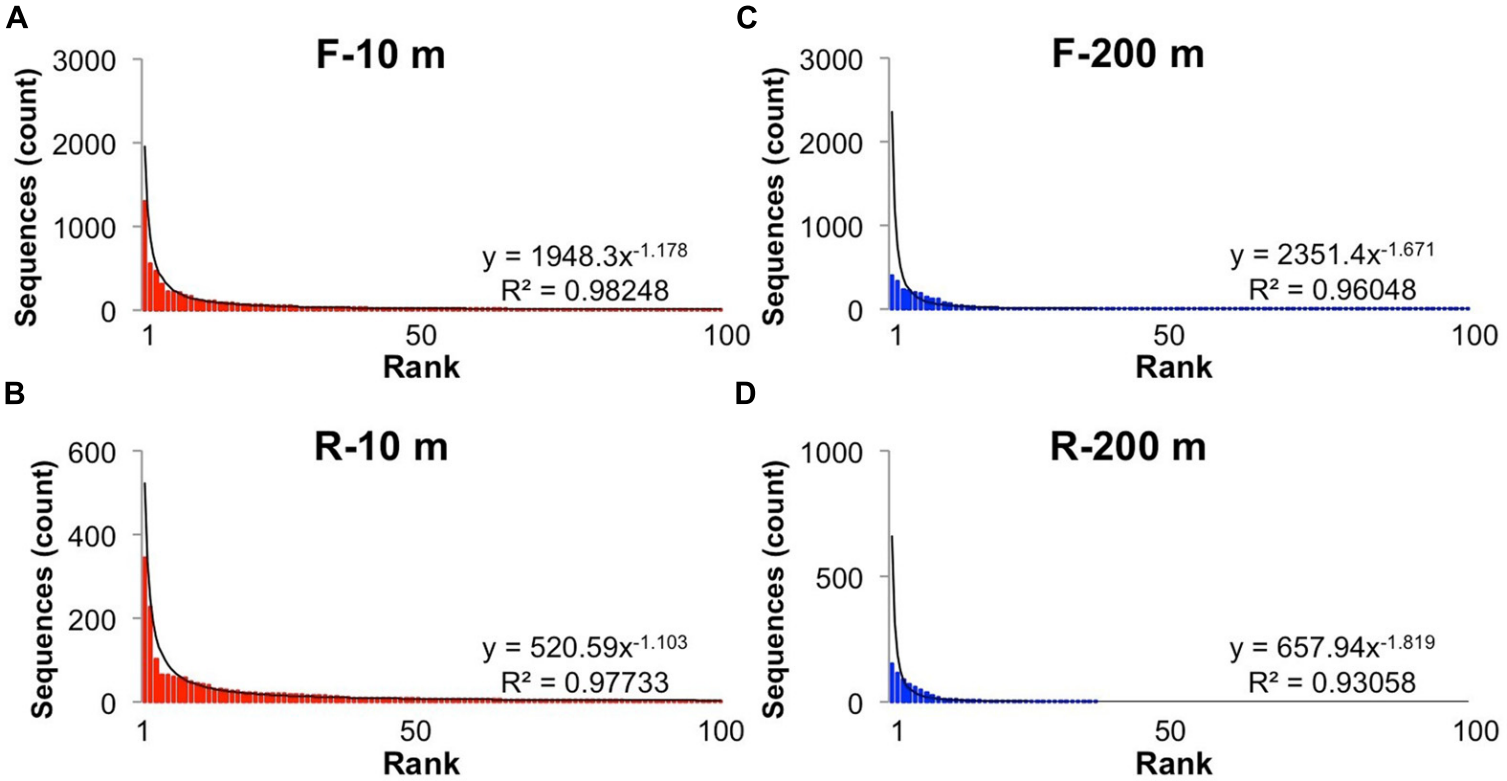
**Rank-abundance distribution of operational taxonomic units (OTUs)**. Rank abundance distribution of the most abundant (up to 100) OTUs (95% similarity) for the forward and reverse primers and each sample: **(A)** Forward primers, 10 m; **(B)** Reverse primers, 10 m, **(C)** Forward primers, 200 m; **(D)** Reverse primers, 200 m. The solid line shows the power-law curve fitting the rank-abundance distribution of phylotypes. Equations and adjusted R^2^ are also shown for each curve.

Reads longer than 200 bp from each bin were clustered into OTUs with more than 95% sequence similarity, and ranked to show the relative abundance of each gokushovirus taxon in our dataset (**Figure 2**). The rank-abundance plots display a similar trend for each primer bin, with a few dominant genotypes and a long tail of doubletons and singletons. The rank abundance distribution of genotypes was approximated by a power-law function with *R*^2^-values >0.95. In contrast, there were fewer OTUs in the 200-m samples, and the distribution was less well described by a power-law function. Phages and their hosts usually follow a power-law rank-abundance distribution (Edwards and Rohwer, 2005; Hoffmann et al., 2007; Suttle, 2007). Possible explanations for environmental phage genotypes following a power-law distribution (Edwards and Rohwer, 2005) are that multiple viruses compete for the same hosts or that each virus is specific for one host but the hosts compete for resources. In the first scenario, the most abundant viruses are the most successful at finding and infecting their hosts; hence, every round of infection produces more of that virus. In the second situation, the host that gets more nutrients divides more rapidly resulting in more available hosts for the viruses. In this case, the most abundant viruses are the ones that infect the most abundant bacteria.

**FIGURE 3:**
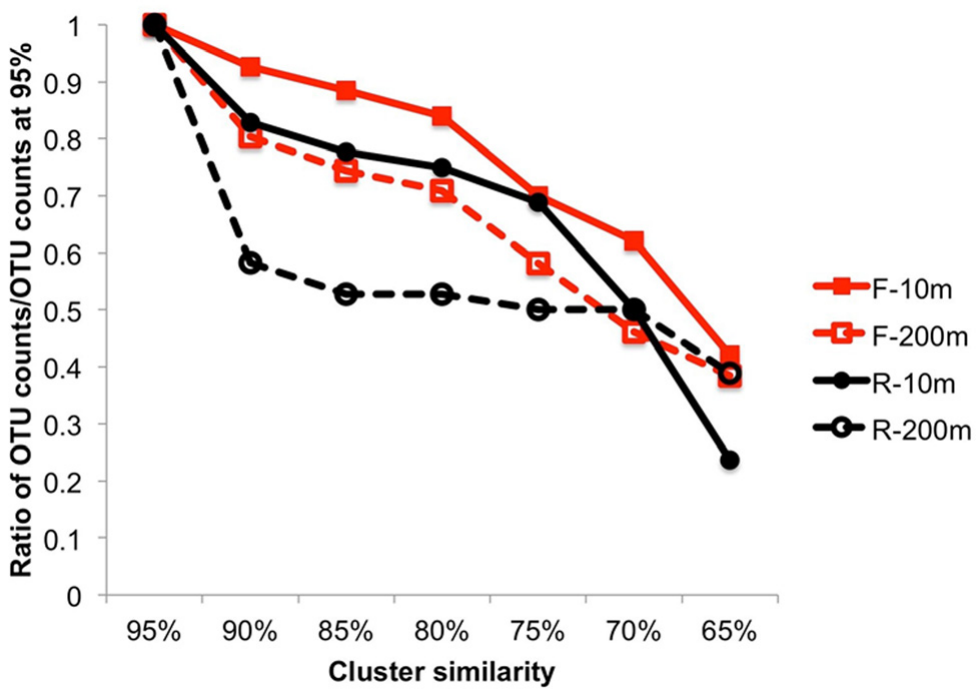
**Cluster analysis from 95 to 65% similarity showing the higher similarity within the end of the amplicon sequences and a lower similarity within the beginning of the amplicon sequences**. The ratio is expressed as the number of OTUs/number of OTUs at 95%.

The overall sequence similarity within primer bins was compared by clustering the sequences using multiple similarity thresholds (**Figure 3**). Both F and R sequences were more similar to each other at 200 m than at 10 m. This was particularly true for the R sequences (3′ end) for which a decrease in the similarity threshold from 95 to 90% resulted in 41.7% fewer OTUs at 200 m, indicating that many of the sequences were similar and suggesting recent infection events. The 3′ end of the PCR product is more conserved, which can explain the higher similarity among OTUs in the reverse primer bins. For the other samples, the decrease in OTUs was steeper when the similarity threshold was below 80%, indicating the sequences were more diverse.

**FIGURE 4:**
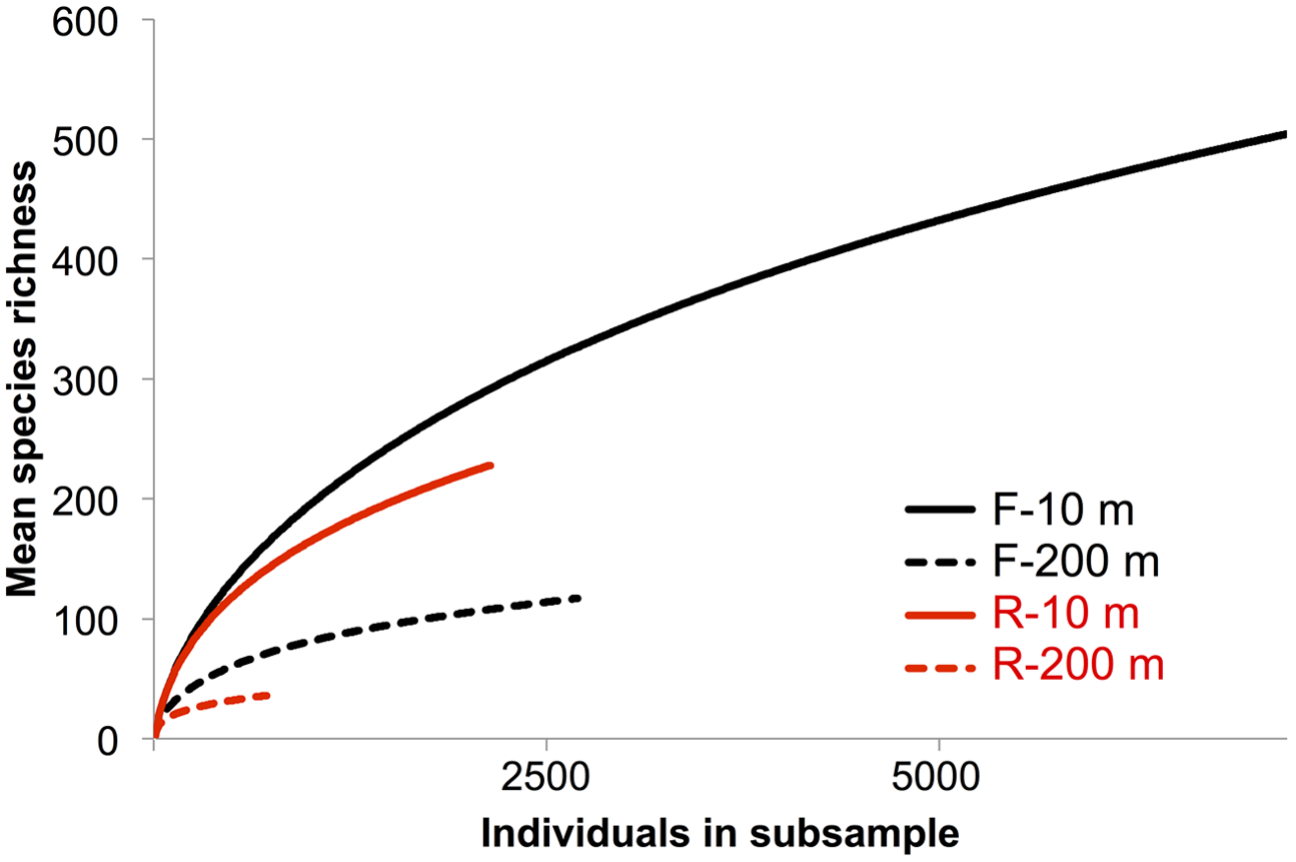
**Rarefaction species richness curves**. Rarefaction curve analysis to infer the number of observed virus phylotypes present within each gene-specific primer bin. Each curve denotes the number of observed OTUs as a function of the number of sampled reads. Reads were clustered into OTUs based on a 95% similarity threshold.

Rarefaction curves did not plateau (**Figure 4**), indicating that the sequencing depth was inadequate to capture the entire gokushovirus community. Based on the number and frequency of each OTU, the total richness was estimated to be between 355 and 504 OTUs at 10 m, and between 49 and 189 OTUs at 200 m (**Table 2**). Therefore, between 65 and 62% of total gokushovirus amplicon richness in SI (**Table 2**) was estimated to be captured using the F primers at 10 and 200 m, respectively. Since most reads were not singletons (e.g., only 2.9% of singletons for the F primers), the richness that was not captured likely comprised rare genotypes. Usually, environmental samples are dominated by relatively few genotypes and many more low-abundance ones that account for most of the genetic diversity (Hoffmann et al., 2007; Suttle, 2007). Moreover, abundant and rare genotypes can be temporally and spatially dynamic with a rare genotype being dominant in a different environment or when conditions change (Sogin et al., 2006; Huse et al., 2010; Gobet et al., 2012; Shade et al., 2014).

**Table 2:** Observed and estimated richness and diversity indices.

	Primer	Observed richness	Estimated richness (*S*_chao_)	Shannon diversity index
10 m	F	504	786±54	3.93
	R	228	355±36	3.87
200 m	F	117	189±31	3.03
	R	36	49±10	2.50

*The richness is expressed in numbers of OTUs defined at 95% identity.*

Care must be taken in interpreting diversity estimates calculated from the data. First, evenness can be influenced by the use of MDA, which can unevenly amplify the initial template (Dean et al., 2001), be biased toward small circular ssDNA molecules and form chimeras (Binga et al., 2008; Polson et al., 2011). Second, the PCR products were pooled from multiple months, which could have affected the evenness observed. For example, if one time point was dominated by a single genotype, it may be highly represented in the pooled sample, even if it was in relatively low abundance at other times. In contrast, a sample with many genotypes at similar abundance will have a lower concentration of each genotype that will be further diluted in the mix. Despite these caveats, and even though temporal variation is integrated across samples, it is reasonable to assume that the observed differences in diversity estimates between the pooled 10 m and 200 m samples is valid. Moreover, because temporal variation is integrated across dates, differences in overall richness are unlikely to be the result of among sample variation; hence, the 4–10-fold higher richness observed in the 10-m samples relative to the 200-m samples (**Table 2**) is likely real. Consistent with this observation, Shannon indices are also higher for the 10-m samples (4.21 and 4.05) than for the 200-m samples (3.18 and 2.54; **Table 2**), reinforcing observations of lower prokaryotic diversity in the anoxic versus oxic waters of SI, with a Shannon index of 0.87 vs. 1.15 for archaea and 0.39 vs. 3.15 at 215 m and 10 m, respectively, (Zaikova et al., 2010).

### Seasonal Variation of Gokushoviruses in the Saanich Inlet

In a previous study Labonté and Suttle (2013a) used RFLP analysis to select MCP amplicons for sequencing from SI, SOG, and the GOM. For SI, RFLP analysis of 180 clones from PCR products spanning nine samples resolved 19 unique bands. More bands were observed in spring and summer, when the bacterial abundance is higher and the water column becomes increasingly stratified, while fewer bands were observed in the fall and winter, when bacterial abundance is lower after deep water renewal. Amplicon 454 sequencing recovered 15 of the 19 sequences from SI associated with the RFLPs (**Table 3**). All four sequences associated with a specific RFLP that were not recovered in the 454 data were found only once, and consequently may have been absent from the 454 data. Since both methods used different DNA preparations, the absence of these sequences may have resulted from MDA or PCR biases. It is also possible that the 454 sequencing was not deep enough, as alluded to in the rarefaction curves that did not plateau (**Figure 4**). A lack of sequencing depth could also explain why most of the RFLP sequences were only found with either the F or R primer, but not both. Nonetheless, the richness recovered was much higher using 454 sequencing than by RFLP analysis.

**Table 3:** Recovery of RFLP Saanich sequences within the amplicon deep-sequencing database.

RFLP sequence	Apr 08	Jan 09	Mar 09	May 09	Jul 09	Aug 09	Nov 09	OTU	Frequency of OTU	Frequency (%)	Sample
**SI-01**											
**SI-02**								DMER9	1/7195	0.01	F-10 m
**SI-03**												
**SI-04**												
**SI-05**								DBSZ3	1/7195	0.01	F-10 m
**SI-06**								D5JJE	2/2135	0.09	R-10 m
**SI-07**								ENNJ4	60/2135	2.81	R-10 m
**SI-07**								DCFCF	1/2135	0.05	F-10 m
**SI-08/SI-09**								DV3J0	1/7195	0.01	F-10 m
**SI-10**								D0YTA	20/7195	0.28	F-10 m
**SI-11**								EW8A2	57/2135	2.67	R-10 m
**SI-12**								C3HEQ	118/7195	1.64	F-10 m
**SI-13**	120							DNX08	64/2135	3.00	R-10 m
**SI-13**	120							F9ET8	49/2687	1.82	F-200 m
**SI-14**								BOBYV	1/7195	0.01	F1-10 m
**SI-16**								D0O8I	1/7195	0.01	F1-10 m
**SI-17**	120											
**SI-18**								EKHXK	5/7195	0.07	F-10 m
**SI-18**								D3RZ4	6/2135	0.28	R-10 m
**SI-SOG-19**								C4GME	3/2135	0.14	R-10 m
**SI-SOG-19**								DSP87	4/7195	0.06	F-10 m

*A total of 15 out of 19 sequences (shown in gray) were sequenced again with amplicon deep sequencing. The seasonal variability of each of these OTUs is represented by dark boxes in the sample months from which they were sequenced. SI, Saanich Inlet; SOG, Strait of Georgia. SI-08 and SI-09 are combined as they were >95% similar. The SI sample depth is indicated if not 10 m.*

In contrast to the SI results, none of the 13 sequences from the GOM and only two out of 12 sequences from the SOG (SOG3-31 and SOG4-29) that were associated with RFLPs, were recovered in the 454-sequencing data. The SOG sample comprised a mixture of 85 virus concentrates from the SOG and surrounding inlets, including SI. The fact that only two sequences were recovered in the 454 data suggests that gokushovirus sequences display a high degree of endemicity. In contrast, studies on the portal protein from myoviruses (Short and Suttle, 2005; Sullivan et al., 2008), and DNA polymerase B from podoviruses (Breitbart et al., 2004; Chen et al., 2009; Labonté et al., 2009; Huang et al., 2010) and phycodnaviruses (Short and Suttle, 2002) have recovered identical or nearly identical sequences from very different environments.

### Phylogenetic Relationships Among Gokushovirus MCP Sequences from Saanich Inlet

**FIGURE 5:**
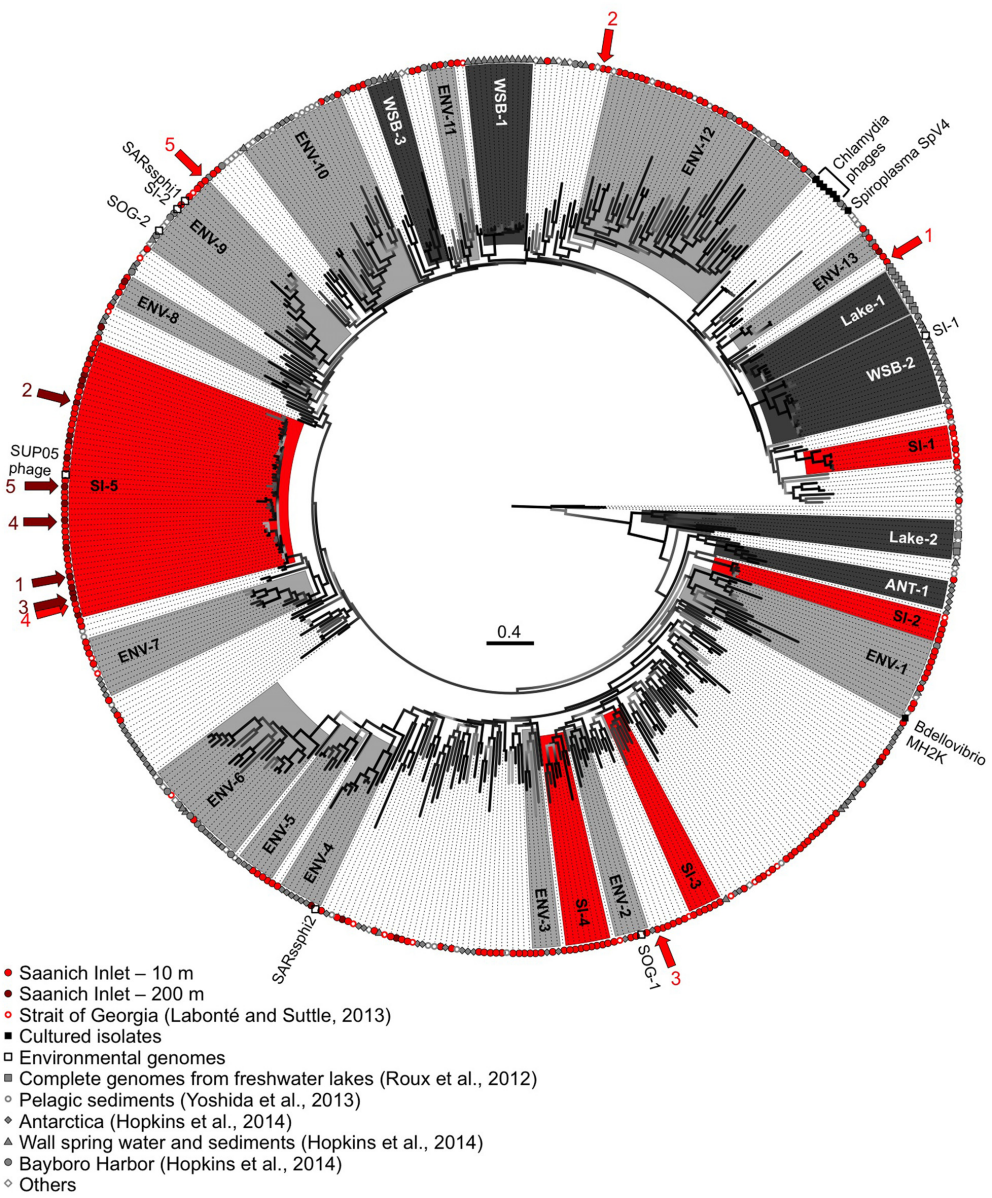
**Genetic relatedness of the gokushovirus major capsid protein (MCP) from SI**. Unrooted phylogenetic analysis (maximum likelihood; HKY85 model; aLRT probabilities) of PCR products from SI (10 m in red, 200 m in dark red). Bootstrap support is represented by a gradient from light gray to black with black representing supported groups with ≥90% support. Red, dark gray, and light gray shaded phylogenetic groups represent well-supported clades of gokushoviruses with more than five sequences from SI only, a single environment, or multiple environments, respectively. The five most abundant sequences from 10 and 200 m are indicated by the red and dark red arrows, respectively, with the ranking indicated by the associated numbers. The scale bar represents 0.4 nucleotide changes per site.

The phylogenetic relationships among MCP sequences from SI were assessed in relation to MCP sequences from isolates and of other environmental PCR amplicons. The sequenced isolates included phages infecting the parasitic bacteria *Chlamydia* sp., *Bdellovibrio bacteriovorans,* and *Spiroplasma melliferum*; of these only MH2K that infects *B. bacteriovorans* has close marine relatives (**Figure 5**). Marine *Bdellovibrio*-and-like-organisms (BALOs) are commonly found in marine environments and parasitize *Vibrio* sp. (Martin, 2002); hence, viruses similar to MH2K could be infecting marine BALOs. In addition to isolates, phage sequences infecting uncultivated SUP05 from SI obtained using single-cell genomics were also included (Roux et al., 2014), as were MCP sequences from other studies that used metagenomic (Roux et al., 2012b; Yoshida et al., 2013) or targeted amplification approaches (Hopkins et al., 2014).

The majority of the sequences fell within supported (≥90% bootstrap support; ≥4 sequences) phylogenetic groups with four or more sequences (**Figure 5**), although sequences also fell outside these clades. Gokushovirus genomes assembled from metagenomics or single-cell genomics were representative of the SI-5, ENV-2, ENV-4, ENV-9, and WSB-2 clades. Many groups comprised sequences that were location specific (red boxes for the SI groups, dark gray for other environments), indicating that gokushoviruses infect endemic bacteria, and congruent with previous studies that suggest a biogeographic separation of gokushoviruses coupled to specific hosts (Labonté et al., 2009; Tucker et al., 2011). However, many groups contained sequences from multiple environments (light gray boxes on **Figure 5**), suggesting that some gokushoviruses infect widely distributed hosts. In contrast to the 43 unique sequences that were recovered from 77 RFLP patterns observed among 400 analyzed clones (Labonté and Suttle, 2013a), high-throughput sequencing allowed the discovery of five previously unknown groups of gokushoviruses.

Phylogenetic analysis of the 3′ end of the gene encoding the MCP (**Figure 5**) revealed that group SI-5 contained most of the sequences from 200 m, as well as some from 10 m that shared >90% nucleotide identity, but none from other locations. Although all the sequences could not be aligned with the 5′ end of the sequences, members of the SI-5 clade shared >80% pairwise identity with the 5′ end supporting the close phylogenetic relationship among these sequences. The five most common sequences from 200 m, and one of the five most common ones from 10 m, fell within the SI-5 clade (**Figure 5**). Based on the RFLP pattern, sequence SI-13 from an April 2007 anoxic-zone sample from 120 m belonged to the SI-5 group, and was also the fourth most abundant sequence in the 10 m deep-sequencing data. Some of the temporal changes in the gokushovirus phylotypes, such as the presence of RFLP sequence SI-13 in the anoxic zone and 10 m sample in April 2007 likely resulted from changes in the bacterial community. These results agree with a metagenomic study of four aquatic ecosystems, in which the dominant viral taxa persisted over time, while the relative abundances of rare ones constantly changed (Rodriguez-Brito et al., 2010). It has been hypothesized that microbial and viral taxa continuously replace each other in a ‘kill-the-winner’ manner, maintaining stable metabolic potential and species composition (Rodriguez-Brito et al., 2010). The bacterial community of SI is dynamic and varies based on changing levels of oxygen-deficiency in the water column throughout the year (Zaikova et al., 2010). As SUP05 dominates in the anoxic zone of SI (Zaikova et al., 2010), Group SI-5 sequences are likely from viruses that infect SUP05. In contrast, viruses infecting more ephemeral taxa likely belong to phylogenetic groups with fewer representative OTUs.

The short genetic distance among sequences in Group SI-5 compared to other clades is consistent with a recent infection event. The presence of sequences from both 10 and 200 m suggests that the event occurred in the fall, during deep-water renewal. Interestingly, gokushovirus sequences associated with single-cell genomes from uncultured SUP05 bacteria, found in marine oxygen minimum zones (Roux et al., 2014), fell within the SI-5 gokushoviruses. SUP05 is the most abundant group of bacteria in the anoxic layer of SI (Zaikova et al., 2010), and it seems likely that SI-5 gokushoviruses infect, and are important agents of mortality for SUP05 bacteria and their relatives.

In contrast, the most common sequences from the 10-m sample fell within unsupported groups containing less than five genetically distinct OTUs. An abundant sequence suggests the occurrence of a recent lytic event or an abundant host. Since many sequences did not fall into any phylogenetic group (**Figure 5**), gokushoviruses likely infect a wide range of bacterial taxa, as suggested with other virus groups, where a wider genetic diversity implies a wider diversity of hosts organisms that are infected (Filée et al., 2005; Clasen and Suttle, 2009). Also, the lower richness of gokushoviruses at 200 m than at 10 m in SI (**Figure 4**; **Table 2**) parallels the differences in bacterial richness between depths (Zaikova et al., 2010), suggesting more potential hosts in the oxic than in the anoxic zone. However, the high similarity of the sequences that clustered with the SUP05 phages, and the specific geographic distribution of gokushovirus sequences, suggest that each gokushovirus has a narrow host range.

In general, a power-law distribution of viral taxa indicates an environment in which viruses infect the competitively dominant hosts (Hoffmann et al., 2007). The viral taxa at 200 m were not as well described by a power-law function as those at 10 m (**Figure 2**), likely because there were fewer sequences and the sequences were more similar to each other. Because the five most abundant sequences at 200 m were in group SI-5 and the frequency distribution of the dominant taxa did not fit a power-law function, it is likely that most sequences from 200 m were the result of a recent lytic event of cells within the SUP05 group, as suggested in Roux et al. (2014). Phenomena that could explain the presence of similar sequences at 10 and 200 m are the yearly deep-water renewal and the sinking and resuspension of viral particles. Deep-water renewal does not result in complete mixing of the water column. Rather, the oxygenated water flowing into SI is denser than the basin water; therefore, it sinks and displaces deep-basin waters upward (Anderson, 1973). An alternative hypothesis is that viruses which sediment on particles during stratification are transported upward during renewal.

No marine gokushovirus has been isolated so far, but they likely infect a wide range of hosts throughout the water column. A 16S ribosomal RNA gene survey from SI (Zaikova et al., 2010) revealed that Bacteroidetes, δ-proteobacteria (Nitrospina), Actinobacteria (Microthrix), and Verrucomicrobia were more abundant at 10 m than at 200 m, and are potential host taxa. Prophages with a similar genome organization to gokushoviruses have been found in the genomes of bacteria from the phylum Bacteroidetes (Krupovic and Forterre, 2011), supporting the idea that these bacteria may be hosts for gokushoviruses in SI.

This study demonstrated that the genetic richness of gokushoviruses was much higher in the oxic (10 m) than anoxic (200 m) layers of SI, and that a power-law function better described the taxonomic distribution of gokushoviruses at 10 m than 200 m, reflecting the bacterial diversity through the redoxcline. Finally, the presence of very similar viruses at 10 m and 200 m is likely due to deep water renewal or potentially biomass sinking from the surface. These results suggest that gokushoviruses infect a wide range of hosts, but that the host range of an individual genotype is narrow.

## Conflict of Interest Statement

The authors declare that the research was conducted in the absence of any commercial or financial relationships that could be construed as a potential conflict of interest.

## Footnotes

1 http://tree.bio.ed.ac.uk/software/figtree/

2 http://dx.doi.org/10.5061/dryad.7gg25
